# Supplementary material for: Single-cell and single-nucleus RNA-sequencing from paired normal-adenocarcinoma lung samples provide both common and discordant biological insights
Source: PLoS Genet. 2024 May 30;20(5):e1011301. doi: 10.1371/journal.pgen.1011301 (PMC11166281; doi:10.1371/journal.pgen.1011301)
Supplement: Attachment — Submitted filename: Renaut_Bosse_response_v26_04_2024.docx [file pgen.1011301.s013.docx]

Quebec, April 30^th^, 2024

Yan Tang, Guest Editor

David Kwiatkowski, Section Editor

PLOS Genetics

Dear Drs. Tang and Kwiatkowski,

Please find attached our revised manuscript entitled ‘Single-cell and single-nucleus RNA-sequencing from paired normal-adenocarcinoma lung samples provides both common and discordant biological insights’ (PGENETICS-D-23-01385).

We would first like to thank the reviewers for taking the time to evaluate our manuscript. We made all efforts to address their comments and provide a point-by-point response below. The revised manuscript includes a better contextualization of our work in relation to reference studies provided by the reviewers; an extensive analysis of gene expression differentiation; H&E staining to confirm that snRNA-seq portrays a more biologically representative view of lung tissue; more extensive analyses of the effect of immune depletion; and numerous other minor changes to streamline the narrative. During this process, we have produced one extra figure in the main text in addition to minor modifications to the original figures, as well as eleven supplementary figures and tables.

We hope that you will find these revisions satisfactory. We thank you for your consideration as we await your editorial decision.

Sincerely yours,

Yohan Bossé, Ph.D.

Professor, Laval University

Department of Molecular Medicine

Canada Research Chair in Genomics of Heart and Lung Diseases

Institut universitaire de cardiologie et de pneumologie de Québec – Université Laval

Pavillon Marguerite-d'Youville, Y2106

2725 chemin Sainte-Foy

Québec (Québec), Canada, G1V 4G5

Tel: 418-656-8711 ext. 3725

email: yohan.bosse@criucpq.ulaval.ca

**Editors**:

**Please be aware that our data availability policy requires that all numerical data underlying graphs or summary statistics are included with the submission, and you will need to provide this upon resubmission if not already present.**

Done

**While revising your submission, please upload your figure files to the Preflight Analysis and Conversion Engine (PACE) digital diagnostic tool. PACE helps ensure that figures meet PLOS requirements.**

Done

**Reviewer #1**:

**In this manuscript, the authors compared single-cell RNA sequencing (scRNA-seq) and single-nuclei RNA sequencing (snRNA-seq) of paired normal-adenocarcinoma human lung samples to explore the similarities and differences in cell types, immune landscapes, and other aspects. The experimental design is appropriate and the technical content is high, providing valuable practical and academic insights for the field of medicine. However, the limitations of this paper lie in its purely descriptive nature and the lack of comprehensive details in result interpretation. Here are a few suggestions for improving the manuscript.**

**Question 1: In main text, article only provides explanations for Figures 1A and 1B. Please provide descriptions for all the main figures would enhance the clarity of the manuscript.**

Each panel from Figure 1 is now described in the revised manuscript (Lines 133-146). In addition, we have made minor adjustments to make sure that each figure and panel are cited in the main text.

**Question 2: Please standardize the sequential letter format in the main figure of the article.**

We have made the necessary corrections. Thank you for noticing.

**Question 3: Line 363, please ensure consistent spacing between numbers and units throughout the document.**

Done

**Question 4: Whether an attempt to perform integrated analysis of Cell and Nucleus datasets could be performed, which may provide a more comprehensive understanding of whether the differences between scRNA-seq and snRNA-seq are solely due to variations in cell proportions.**

Please note that a UMAP integrating all *Cell*, *Immune-depleted* *cell* and *Nucleus* datasets was available in Fig. S5 of the original manuscript. However, we now introduced this figure earlier in the result section (Figure S1, cited at L152 in the revised manuscript).

In addition, we now provide a more comprehensive analysis of *Cell* and *Nucleus* datasets beyond variations in cell proportions. This analysis of cell type specific gene expression is described in the Methods (L627-650), Results (L243-269) and Discussion (L401-411). We have also added one new figure in the main text (Figure 8), 7 supplementary figures (Figure S3-5, S8-11) and 5 supplementary tables (Table S4-S8).

**Question 5: Please analyze cellular communication and other relevant factors using Immune-depleted datasets to better elucidate how Immune-depleted cell datasets can mitigate the differences between Cell and Nucleus datasets.**

We provide new analyses to address this question. We added one supplementary figure (Fig. S11), which we describe in the Results section (L306-301). In addition, we moved one supplemental figure into the main Figure 10 (panel F), and revised lines 318-320 of the Discussion.

**Question 6: The “Cell composition differs from Nucleus in Normal lung tissue” part mentions that the cellular landscapes differ greatly between Cell and Nucleus datasets, and proposes that in the context of lung adenocarcinoma and patient-matched normal samples, Nucleus provides a cell populations more closely matching tissue content. Whether HE staining or other experimental results could be provide to validate this conclusion?**

H&E staining of paired normal-adenocarcinoma lung tissues are provided in the revised manuscript (see Figure S4A-B, Results at L171-172, Methods at L461-476, Discussion at L352). As expected, histologic evaluation shows H&E sections that are largely dominated by epithelial cells compared to immune cells, a pattern consistent will the *Nucleus* dataset.

**Question 7: The “The cellular transition to lung adenocarcinoma” part mentions that there is inconsistency in the transition from normal to tumor in some immune cells between Cell and Nucleus datasets, Whether immunohistochemical staining or other experimental results could be provided to confirm this conclusion and compare the accuracy of the two sequencing methods?**

We first modified the panel B of Figure 7 in order to show results for each patient, instead of averaging all patients. This way, it now becomes apparent that there are no consistent pattern between scRNA and snRNA in the transition from Normal to Tumor, and we have modified the text accordingly (L230-232).

In addition, “When analysing more specifically interstitial macrophages (level 4 annotation), we confirmed a consistent augmentation in Tumor samples in *Cell* and *Nucleus* that was corroborated by immunohistochemical staining (Fig. S4C)” (L231-233).

We found “a discordant pattern for Monocytes” (L229) and cannot conclude which method is closer to the real cellular composition of tissues. Yet, we caution that “For Immune cells, patterns were harder to interpret given the small number of immune cells in the *Nucleus* dataset.” (L226-227).

**Question 8: In “The effect of immune depletion on Cell sequencing” part, the impact of gene expression site on the results whether has been considered in the evaluation of stress-related genes?**

We are unsure about what the reviewer means by “gene expression site”. In Figure 10E we compared the stress-related gene signature across methods (*Cell*, *Nucleus*, *Immune-depleted cell*) and tissue types (Normal lung and Tumor). We have also evaluated whether the stress-related genes are associated with the tumor location. As indicated in Table S1, the tumors were located in the left upper lobe for two patients and in the right upper lobe for the other two patients. We have repeated Figure 10E, but stratified by tumor location (Left Upper Lobe vs Right Upper Lobe). As shown in the figure below, there is no effect of the tumor location. We hope this answers the question.

**Reviewer #2**:

**Renaut et al. performed a head-to-head comparison between scRNA-seq and snRNA-seq in matched normal-adenocarcinoma human lung samples. They obtained more than 160,000 cells/nuclei from 4 patients and detected a significant difference in cell type proportions between scRNA-seq and snRNA-seq. They also revealed the cellular and molecular transition that occurs from normal lung tissue to adenocarcinoma, and evaluated the commonality and discordance in the biological insights gained from cells versus nuclei. Particularly, the ligand-receptor interactome landscape of lung adenocarcinoma varied significantly between scRNA-seq and snRNA-seq. Finally, they concluded that scRNA-seq is fit for the immune landscape mapping of lung adenocarcinoma, while snRNA-seq may capture more reasonable cell type proportion.**

**This is a comprehensive benchmarking study for the comparison between scRNA-seq and snRNA-seq in matched normal-adenocarcinoma human lung samples. The data provides a useful resource for the related studies, and the findings can help researches to choose appropriate method when dealing with similar issues. Several concerns remain to be addressed, as shown below.**

**Major:**

**1. In the introduction part, the authors should cite the previous study about liver scRNA-seq and snRNA-seq comparison, since this study also obtained similar conclusion (Single-Cell, Single-Nucleus, and Spatial RNA Sequencing of the Human Liver Identifies Cholangiocyte and Mesenchymal Heterogeneity, https://doi.org/10.1002/hep4.1854).**

Thank you for pointing this excellent manuscript comparing scRNA-seq and snRNA-seq in human liver which we now mention in the introduction (L95-96) “*Combining scRNA-seq and snRNA-seq technologies from matched samples has been shown to better capture cell heterogeneity and produce a more comprehensive cell map of healthy human liver”.*

We also added these sentences in the Discussion:

“*Andrews et al. compared cells and nuclei of matched healthy human liver and concluded that cell-type frequencies were distorted in scRNA-seq”* (L347-348)

*This is consistent with the recommendation of using scRNA-seq to investigate immune populations in the human liver”* (L435-436).

**2. Considering the more dropout rate and less transcript abundance in snRNA-seq, it is maybe not appropriate to annotate the snRNA-seq dataset with the single-cell based reference, HLCA, though it is maybe fit for scRNA-seq dataset. As their results shown that more unclassified identities are presented in the snRNA-seq dataset. Thus, unsupervised classification is also needed to provide more accurate cell type annotation, and a separate annotation of scRNA-seq and snRNA-seq datasets is also a good way to double check the accuracy of the cell identities.**

Please note that each of the 24 samples we sequenced was annotated separately. The weight of each gene into the principal components (top 50 PCs) was defined by the HLCA reference and as such, each sample was integrated into the same 50-dimensional space as the reference. We acknowledge in the Discussion the advantages of this approach, but also the limitations (L362-383). In addition, we now mention how in a recent paper, Trinks and colleagues (Cell. Oncol. 2024) used a similar statistical approach to annotate their snRNA-seq dataset comparing patient-matched lung adenocarcinoma samples (see L365-L367).

In Figure S2 (discussed at L364), we present the result of a gene marker-based annotation, as a way to double check the accuracy of the cell identities defined through the reference-based approach. This supports the idea that it performed as well as a marker-based approach, at least at the coarsest annotation level.

Finally, we now present some complementary results, using Principal Component Analyses of the expression of markers genes typically used to distinguish the main cell types, to double check that the reference-based annotation of the samples matches with the information provided by gene markers (Figure S10). Nevertheless, much like in the reference-based approach, markers genes were less efficient in distinguishing cell types in the *Nucleus* samples. We present these results at L258-269 and discuss this at L362-383.

**3. The author should also check whether their findings are shared by all the four patients or biased by individual patient, such as the results in Figure 3 and 5.**

The variation in cell types proportions among patients from Figure 3 and 5 are presented in Figure 4 (panels A & D) and 6 (panels A & D). We now provide this information per patient in supplemental UMAPs as well (Figure S3 & Figure S5, mentioned at L170 and L205, respectively).

**4. In the CNV analysis, the authors stated that other cell types rather than AT2 exhibited higher CNV scores. What are the normal reference cell types for inferring the CNV scores, as the normal reference cell types can greatly affect the result. I do not think it is appropriate to use immune cells to perform the CNV analysis, because malignant cells mainly originate from epithelial cells, thus the author should use normal epithelial cells as reference, including each normal epithelial cell type.**

We have revised our approach and have modified the Methods (L600-612), Results (L234-L241) and Discussion (L388-391) according to the reviewer’s suggestion. In addition, given that we do not quantify a CNV score in all cell types anymore, but solely in epithelial cells, we have removed the CNV results from figure 6 and instead, now present it as a panel in Figure 7 (transition from Normal to Tumor) solely for epithelial cells.

**5. The authors didn’t identify the malignant cells in tumor samples neither in ‘Cell dataset’ nor ‘Nuleus dataset’, which should be one necessary step for analyzing tumor samples.**

Delineating cancer from non-cancer cells in single-cell studies remains an important challenge in the field. CNV analyses are one way to identify putative malignant cells, but there are, to the best of our knowledge, no standardized method to objectively dichotomize cancer vs non-cancer cells.

Nevertheless, we attempted to classify cells as malignant based on the premise that malignant cells have an epithelial origin, a high CNV score and are more difficult to annotate into specific cell (epithelial) types. Consequently, we defined malignant cells as epithelial cells with CNV score in the top quintile and a cell type annotation (level 3) score in the bottom quintile. This allowed an objective comparison between methods and patients. We have described this in the Methods (L607-612), Results (L239-241) and Discussion (L390-391).

**6. For cellular transition analysis, the authors mentioned that ‘For immune cells, patterns were harder to interpret given the small number of immune cells in the Nucleus dataset’. As shown in Figure 6B and F, the number of immune cells in ‘Nucleus dataset’ is bigger than that in ‘Cell dataset’, therefore, the interpretation is not accurate.**

We believe that the reviewer misread the Figure; there are more immune cells in the “*Cell* dataset” (n=20410, Figure 6B) than the “*Nucleus* dataset” (n=5764, Figure 6E of the revised manuscript).

**7. The authors compared the cell composition and cell communication difference between ‘Nuleus dataset’ and ‘Cell dataset’. The authors also should identify the molecular differences between the two datasets for specific cell types, which might identify some cell-type shared or specific molecular differences, giving some clues for studying biological insights difference between the two datasets.**

See response to point #4 of reviewer #1.

We now provide a more comprehensive analysis of *Cell* and *Nucleus* datasets beyond variations in cell proportions. This analysis of cell type specific gene expression is described in the Methods (L627-650), Results (L243-269) and Discussion (L401-409). We have also added one new figure in the main text (Figure 8), 7 supplementary figures (Figure S3-5, S8-11) and 5 supplementary tables (Table S4-S8).

**Minor:**

**1. In the first sentence of the Abstract section, snRNA-seq should be ‘single-nucleus’ not ‘single-nuclei’.**

Done

**2. In the first sentence of the Introduction section, single-cell transcriptomics should not be short for ‘scRNA-seq’, single-cell RNA-sequencing is more appropriate. Likewise, the full name for snRNA-seq should also be mentioned when it appears for the first time (Page 1, Line 67).**

Done

**Reviewer #3**:

**This study compares single-cell RNA-sequencing (scRNA-seq) and single nuclei RNA-sequencing (snRNA-seq) in matched normal-adenocarcinoma human lung samples with a total of 160,621 cells/nuclei. They mainly find that while scRNA-seq captures more immune cells in non-tumor lung and snRNA-seq captures more epithelial cells, the two methods show discordant cell type transitions from normal to tumoral tissue.**

**Although this study conducted a relatively wide range of experiments, my main concerns with this manuscript are that the authors did not analyze and discuss the experimental results deeply enough. Moreover, this study did not further explore and elucidate the development of lung adenocarcinoma with significant phenotypic changes and key gene expression changes using single cell and single nucleus sequencing techniques. In fact, as early as 2020, there were several papers that conducted similar experiments and analyses on kidney (PMID: 30510133), PBMCs (PMID: 32341560) and even the same non-small cell lung cancer samples (PMID: 32405060) as this study. As a result, this study does not show enough innovation and powerful insights compared to previous research work.**

We have now included additional analyses and further discussions on the corresponding results in the revised manuscript, and we believe our current experimental design and results go much beyond the complementary work mentioned by the reviewer. Mainly, we now provide an extensive analysis of gene expression differentiation; more extensive analyses of the effect of immune depletion; and numerous other minor changes to streamline the narrative. See also responses to other reviewers’ comments in addition to responses below.

The three articles mentioned by the reviewers are cited in our manuscript. A brief summary of these studies and others is provided in the table below to show how unique is our dataset compared to previous studies.

| **Article**  **PMID** | **Species** | **Tissues** | **Methods** | **Number of cells/nuclei** | **Considerations/Conclusions** |
| --- | --- | --- | --- | --- | --- |
| Wu et al. 2019  30510133 | mouse | kidney | 4 platforms, incl. 10x | 11,391 | -Single-cell was performed by DropSeq, whereas single-nuclei by three methods, including 10x Genomics.  -The design does not allow comparison between single-cell vs single-nuclei on the 10x platform.  -snRNA-seq detect more cell types (reduced dissociation bias), does not impair cell identification, and eliminates stress response genes. |
| Ding et al. 2020  32341560 | human & mouse | Cell lines, human PBMCs, mouse brain | 7 methods, incl. 10x | 92,000 | -Only mouse brain tissues were evaluated by single-nuclei (and single-cell from the same specimens were not available).  -Similarly, for PBMCs, single-cell data were compared across methods, but single-nuclei from PBMCs were not available.  -So, this study does not provide a head-to-head comparison between single-cell and single-nuclei. |
| Slyper et al. 2020  32405060 | human | 8 tumor types, incl. NSCLC | 10x | 216,490 | -This study evaluated tumor-only material (not paired normal-tumor).  -NSCLC samples were profiled by single-cell only (not single-nucleus). |
| Lake et al. 2017  28729663 | mouse | brain | smart-seq | 399 | -Single-nucleus accurately captures cellular identity.  -Cell types identified in cells and nuclei were the same, but at different proportions.  -Gene expression concordance between cells and nuclei is greater for longer genes.  -Mitochondrial respiratory-associated transcripts are almost exclusively detected in cells. |
| Denisenko et al. 2020  32487174 | mouse | kidney | 10x | 77,656 cells & 98,303 nuclei | -There is an underrepresentation of immune cells in snRNA-seq data.  -Cell type composition differs from the original tissue with both single-cell and single-nucleus. |
| Bakken et al. 2018  30586455 | mouse | brain | FACS + RNA-seq | 463 cells & 463 nuclei | -Gene detection is higher in cells than nuclei.  -20.5% of genes are more expressed in cells, and 0.4% are more expressed in nuclei.  -snRNA-seq provides similar resolution for cell type detection compared to scRNA-seq. |
| Andrews et al. 2022  34792289 | human | liver | 10x | 29,432 cells & 43,863 nuclei | -There are several differences between scRNA-seq and snRNA-seq.  -A more complete map of cell types is obtained by combining both scRNA-seq and snRNA-seq.  -Cell type frequencies are distorted in scRNA-seq.  -snRNA-seq detected additional cell subtypes including rare subtypes.  -Important markers of immune cells were absent in snRNA-seq, suggesting that scRNA-seq is more appropriate to study immune populations. |

As we emphasized in our manuscript, the difference between scRNA-seq and snRNA-seq is most likely context-dependent. Consequently, we cannot assume that insights from circulating cells (such as PBMCs) or other solid tissues (such as the kidney, especially from mice) will provide the same information and reach the same conclusion than paired normal-adenocarcinoma human lung samples. Regarding Slyper et al. (2020), they have tested only the tumor of two patients with NSCLC, and not paired normal-tumor lung samples. Note that NSCLC is a broad category of lung cancer, including adenocarcinoma, squamous cell carcinoma and others. The specific histologic subtype is not provided by Slyper et al. (2020).

We added this paragraph in the Discussion:

“*This study has methodological implications as the literature and data comparing scRNA and snRNA are still scarce. Previous studies have compared scRNA and snRNA methods, but data from the same specimens were not necessarily available^11–13^. Head-to-head comparisons with the same specimens were performed using different platforms in mouse brain^15,16^ and with 10x Genomics in mouse kidney^14^. In humans, we are only aware of one 10x study comparing matched scRNA and snRNA from human liver^17^. In the current study, we have both single-cell and single-nuclei on both normal lung and adenocarcinoma samples for all four patients and on the same platform (10x Genomics). Samples were resected in the same hospital and sequenced by the same laboratory. We thus have a unique and much-needed dataset to study the difference between single-cell and single-nucleus RNA-seq. By sharing our data with the scientific community, we aim to stimulate further comparisons between scRNA and snRNA, and allow others to build on our results.*”

**Main concerns:**

**1. Authors should use single cell and single nucleus sequencing techniques to further explore potential cell interaction changes or gene expression changes that may be associated with lung adenocarcinoma through a variety of downstream analytical tasks, such as gene differential expression or gene regulatory network analysis.**

We now provide a more comprehensive analysis of *Cell* and *Nucleus* datasets beyond variations in cell proportions. Gene expression analysis (including Gene Ontology enrichment result and Principal Component Analyses) are described in the Methods (L628-650), Results (L244-269) and Discussion (L379-381, L401-409). We have also added one new figure in the main text (Figure 8), 7 supplemental figures (Figure S3-S5, S8-S11) and 5 supplemental tables (Table S4-S8).

**2. For the multiple experimental results of this manuscript, the author should carry out detailed analysis and discussion, rather than simply display them.**

Analyses, Results and Discussion have been extended with more details in the revised manuscript.

**3. The impact of immune cell depletion on downstream analysis and interpretation is not fully characterized.**

See answer to question #5 of reviewer #1. We did conduct further analyses of the effect of immune depletion. Consequently, we have added one supplemental figure (Figure S11), which we describe in the Results section (L296-301). In addition, we have also moved one supplemental figure into the main Figure 10 (panel F), and revised L332-334 of the Discussion.
